# Bone Metabolism and the c.-223C > T Polymorphism in the 5′UTR Region of the Osteoprotegerin Gene in Patients with Inflammatory Bowel Disease

**DOI:** 10.1007/s00223-016-0192-9

**Published:** 2016-09-17

**Authors:** Iwona Krela-Kaźmierczak, Marta Kaczmarek-Ryś, Aleksandra Szymczak, Michał Michalak, Marzena Skrzypczak-Zielińska, Natalia Drwęska-Matelska, Michalina Marcinkowska, Piotr Eder, Lilianna Łykowska-Szuber, Ewa Wysocka, Krzysztof Linke, Ryszard Słomski

**Affiliations:** 1 Department of Gastroenterology, Human Nutrition and Internal Diseases, Poznań University of Medical Sciences, Przybyszewskiego 49, 60-355 Poznan, Poland; 2 Institute of Human Genetics, Polish Academy of Sciences, Strzeszyńska 32, 60-479 Poznan, Poland; 3 Department of Computer Science and Statistics, University of Medical Sciences, Poznan, Poland; 4 Department of Biochemistry and Biotechnology, University of Life Sciences in Poznań, Poznan, Poland; 5 Department of Family Medicine, University of Medical Sciences, Poznan, Poland; 6 Department of Laboratory Diagnostics, University of Medical Sciences, Poznan, Poland

**Keywords:** Osteoporosis, Inflammatory bowel disease, Osteoprotegerin, *TNFRSF11B* gene polymorphism

## Abstract

Osteoporosis is more frequent in inflammatory bowel disease (IBD) patients. A reduction in bone mineral mass in these individuals is caused not only by inflammatory processes in the bowel, because osteoporosis occurs already in very young IBD patients and in newly diagnosed individuals who have not yet undergone any pharmacological treatment. One of individual determinants of the bone turnover parameters is osteoprotegerin (OPG) encoded by the *TNFRSF11B* gene. The c.-223C > T polymorphism in this gene has been extensively studied in post-menopausal osteoporosis patients. However, no such studies exist for osteoporosis related to IBD. The aim of our study was to determine whether the c.-223C > T (rs2073617) polymorphism in the 5′UTR region of the gene encoding osteoprotegerin is a functional polymorphism which may change the gene expression and resulting OPG levels, and so be associated with osteopenia and osteoporosis, and impaired bone metabolism in Crohn’s disease and ulcerative colitis patients. Our study included 198 IBD patients and 41 healthy controls. Lumbar spine and femoral neck bone mineral density, T-score, Z-score as well as OPG, RANKL, vitamin D, calcium and interleukin 4 and 10 concentrations were determined for all study subjects. Genotyping of the *TNFRSF11B* polymorphic site was performed by restriction fragment length polymorphism technique. Statistical analyses were conducted using Statistica software. Odds ratios, 95 % confidence intervals, and *P* values were calculated using the HWE calculator. Our results did not allow determining an unequivocal association between the polymorphic variants of the *TNFRSF11B* 5′UTR region and a susceptibility to osteoporosis in IBD patients. We have shown, however, that the c.-223T allele was twice as more frequent in Crohn’s disease (CD) patients than among controls (OR = 1.99, *P* *value* = 0.009). Interestingly, average osteoprotegerin levels in CD patients did not significantly differ from those in controls, whereas in ulcerative colitis patients, OPG levels were significantly lower. We have concluded that low OPG levels may be associated with osteoporosis in ulcerative colitis, but it is not correlated with the c.-223C > T polymorphism in the *TNFRSF11B* gene. In CD patients, in turn, we observed increased RANKL levels. Our observations confirm different pathogeneses of Crohn’s disease and ulcerative colitis as well as different molecular backgrounds of osteoporosis associated with these two diseases.

## Introduction

Osteoporosis in inflammatory bowel disease (IBD) patients is more frequent and requires prophylactic, diagnostic and therapeutic measures [1]. This may be due to both undernourishing resulting from the disease process in the digestive system and steroid-based therapy. However, recent reports in the literature point out that a reduction in the bone mineral density (BMD) in IBD patients is not only a consequence of chronic inflammatory processes in the bowel because osteoporosis occurs already in very young IBD patients as well as in newly diagnosed patients who have not yet been administered any drugs [2]. Therefore, studies linking the common pathogenesis of these two diseases are of particular importance.

One of the causes of osteoporosis is imbalance between the osteoblast and osteoclast activities leading to increased bone resorption. Bone metabolism at the osteoclast/osteoblast level is regulated among others by the RANKL/RANK/OPG signalling pathway. This pathway is dependent on pro- and anti-inflammatory cytokines whose activities are altered in IBD patients. The RANKL/RANK/OPG signalling pathway comprises the following factors belonging to the tumour necrosis factor superfamily: receptor activator of nuclear factor kappa B (κB; RANK), its ligand (receptor activator of nuclear factor κB ligand, RANKL) and osteoprotegerin (OPG) [3–6]. RANK is found on the surface of osteoclast precursor cells. The complex RANK-RANKL induces the differentiation of pre-osteoclasts into mature cells and an increase in the osteoclast activity as well as inhibits their apoptosis, which leads to increased bone resorption and the development of osteoporosis [7]. It is known that genetic variants of genes encoding the RANKL/RANK/OPG signalling pathway molecules may affect the development of osteoporosis [8]. It is also known that this signalling pathway participates in the regulation of inflammatory processes. In a meta-analysis of six genome-wide association studies (GWAS) on Crohn’s disease, Franke et al. [9] pointed to the *RANKL* gene, among others, as a new quantitative trait locus (QTL), which may be related to a CD-associated osteoporosis.

One of the genes that determine the parameters of the bone turnover is *TNFRSF11B* encoding osteoprotegerin (OPG). OPG is a cytokine excreted by active osteoblasts. By binding to RANKL, it inhibits its binding with RANK, which in turn leads to the inhibition of the osteoclast maturation pathway. Thus, OPG plays a role of a bone resorption inhibitor. Single nucleotide polymorphisms in this gene have been so far extensively studied in post-menopausal osteoporosis [10–17]. For osteoporosis associated with IBD, such studies do not exist.

The *TNFRSF11B* gene (OMIM: 602643, HGNC: 11909) is localised on chromosome 8 (8q23-24). Its expression starts between the 8th and 9th day of the embryogenesis [18], and it occurs not only in bones but also in tissues of many organs like heart, lung, kidney, liver, thyroid, brain or placenta [19]. Osteoprotegerin belongs to the group of anti-inflammatory cytokines. It also directly regulates bone metabolism. Moreover, many of the known factors influencing the bone metabolism act at least to some extent by regulating the *TNFRSF11B* gene expression [20].

Moschen et al. [21] described IBD-related alterations in the RANKL/OPG system and their association with a lowered BMD. They showed increased OPG concentrations in plasma as well as its increased release from the inflamed large bowel in IBD, pointing to macrophages and dendritic cells as sources of OPG in the large bowel in IBD patients. These studies suggested that osteoprotegerin expression may be the host-protective response which partly compensates for the negative influence of the chronic inflammatory condition on the bone system. They confirmed the key importance of dendritic cells in the regulation of the inflammatory processes in the bowel of IBD patients. The dendritic cells are involved, among others, in the induction and expansion of pathogenic and regulatory T cells in the lymphatic tissue and in inflamed areas.

Other studies have shown that increased bone metabolism may be associated with *TNFRSF11B* sequence variants which downregulate or block the expression of the osteoprotegerin gene [22, 23]. In a recent paper, Xue et al. [24] showed that OPG serum levels were significantly higher in patients with intervertebral disc degeneration and the TC or CC genotype at the c.- 223C > T polymorphic locus (rs2073617) than those carrying the wild-type homozygous (TT) genotype. Earlier, Vidal et al. [25] have proven an association between the c.-223T allele and lowered osteoprotegerin levels in blood serum.

The aim of this study was to determine whether the c.-223C > T polymorphism in the *TNFRSF11B* gene is a polymorphism which may be associated with lower osteoprotegerin concentration in blood serum, lower bone mineral density and the occurrence of osteopenia and osteoporosis in inflammatory bowel disease patients.

## Methods

### Study Subjects

A total of 198 patients hospitalised at the Department of Gastroenterology, Human Nutrition and Internal Diseases of Poznan University of Medical Sciences, because of disease flare between 2011 and 2014 were prospectively enrolled into the study. These patients included 100 individuals with Crohn’s disease (CD; 51 males and 49 females) and 98 individuals with ulcerative colitis (UC; 46 males and 52 females).

The inclusion criteria were as follows: age between 18 and 60 years, diagnosis of IBD based on cross-sectional imaging and/or endoscopy with histopathological confirmation, disease duration over 6 months, reduced BMD measured by dual-energy X-ray absorptiometry (DXA) and/or low-energy fractures in clinical history, lack of any other condition (e.g. rheumatoid arthritis, chronic renal failure), which could affect BMD.

The controls (C) were 41 healthy volunteers (21 males and 20 females) without IBD or other conditions that influence the condition of the bone tissue, and with normal bone mass as confirmed by densitometry.

All individuals gave their written consent to genetic testing and evaluation of chosen biochemical parameters in serum. The study was approved by the Bioethical Committee of the University of Medical Sciences in Poznań, Poland, under Resolution No. 92/09.

### Clinical Examination

Peripheral blood was collected from all participants in order to perform further biochemical and genetic studies. Bone mineral density of the lumbar spine (L1-L4) and femoral neck (FN) of all individuals was measured by dual-energy X-ray absorptiometry (DXA) method with the DPX-Plus device (Lunar). The apparatus was calibrated daily, and the coefficients of variation (CV) for the BMD measurements were 1.25 % for the lumbar spine and 1.80 % for the femur.

The weight, height and age at the densitometric analysis were recorded for each individual. Serum concentrations of osteoprotegerin (OPG) and of total (both free and bound form) receptor activator of nuclear factor (NF)-κB ligand (sRNAKL) were measured by a sandwich immunoassay method with monoclonal antibodies (ELISA test, BioVendor-Laboratorni medicina, a.s., Czech Republic) and microplate reader SunriseTM (Tecan Group Ltd, Switzerland), with a sensitivity of 0.1 pmol/l. The intra-assay variation coefficients were 6.0 % for OPG and 6.5 % for sRNAKL, and the inter-assay coefficients were 7.2 % for OPG and 6.9 % for sRANKL.

Serum calcium levels were determined in all patients (based on reference value ranging from 2.15 to 2.55 mmol/l) using the Roche assay and a Cobas analyser (Roche). The coefficients of variation ranged from 0.8 to 2.5 %. The serum concentrations of 25-hydroxyvitamin D (25(OH)D) were determined by the electro-chemiluminescence binding assay and a Cobas analyser (Roche), with a functional sensitivity of 4.01 ng/ml (CV = 18.5 %).

Serum samples for interleukin measurements were stored at −25 °C until use (on average for 2 months). Concentrations were determined using a quantitative sandwich enzyme-linked immunosorbent assay (ELISA kit, R&D Systems, USA) with monoclonal antibodies specific for interleukin (IL) 4 and 10, and microplate reader SunriseTM (Tecan Group Ltd, Switzerland). The sensitivity was 10 pg/ml for IL-4 and 0.09 pg/ml for IL-10. The coefficients of variation were as follows: for IL-4 intra-assay 4.1 % and inter-assay 5.0 %, and for IL-10 intra-assay 7.0 % and inter-assay 8.2 %.

### DNA Extraction and *TNFRSF11B* rs2073617 Polymorphism Genotyping

DNA was extracted from whole blood leucocytes using guanidine isothiocyanate and phenol–chloroform. Isolates were dissolved in 1×TE buffer and stored at −20 °C until use [26]. The genotyping of the *TNFRSF11B* polymorphism in the 5′UTR region: c.-223C > T (rs2073617), localised 223 bp upstream of the translation initiation site, was performed by the restriction fragment length polymorphism (RFLP) technique. The fragment containing the polymorphism was amplified using the following primers: sense 5′-AGCCGCCTTGTTCCTCAG and antisense 5′-GTCCGGCAACAGGAAGTT (Tm = 57 °C), giving a PCR product of 255 bp [27]. Amplification products were subsequently digested with the *Ksp*AI restriction enzyme (New England Biolabs) which yields fragments of 126 bp and 129 bp for the c.-223T allele. The digestion products were separated by 1.6 % agarose gel electrophoresis.

### Statistical Analysis

The normality of the distribution and the homogeneity of variable variances were verified in the studied groups using Shapiro–Wilk *t* test and Levene’s test, respectively. In the event of non-concordance with two or at least one condition, the nonparametric Kruskal–Wallis *H* test was used to compare the groups. In case of a statistically significant heterogeneity between groups, multiple comparisons were conducted using the Dunn’s test. In order to evaluate an association between qualitative variables (the three study groups versus groups carrying different *TNFRSF11B* gene alleles), Chi-squared test was used. All analyses were conducted using STATISTICA 10.0 software (StatSoft), and the analysis of genotype distribution’s concordance with Hardy–Weinberg equilibrium was performed using the calculator on the http://ihg.gsf.de/cgi-bin/hw/hwa1.pl website [28]. *P* values below 0.05 were considered to indicate statistical significance.

## Results

Basic characteristics of the study subjects are presented in Table 1. All groups contained similar proportions of males and females. The mean age of subjected CD patients was 35 and 39 in UC patients. We observed higher levels of RANKL in sera of CD patients as compared to UC patients (297.24 vs 228.21 pmol/l, *P* value = 0.013), whereas in UC patients the average concentration of RANKL was not significantly different from that found in controls. UC patients, in turn, had the lowest sOPG levels (6.26 pmol/l vs 9.59 pmol/l in controls and 8.65 pmol/l in CD patients, *P* value <0.0001 for both) and the lowest sOPG/sRANKL ratios (0.046 vs 0.051 in CD patients, *P* value = 0.042).

**Table 1:** Study groups basic and clinical characteristics

	CD patients (*n* = 100)	UC patients (*n* = 98)	Controls (*n* = 41)	*P* value for comparisons between groups		
				CD versus C	UC versus C	CD versus UC
Age (years)	35.59 ± 12.79	39.46 ± 14.69	30.37 ± 8.58	ns	*P* = 0.001	ns
Body mass (kg)	63.39 ± 13.71	68.38 ± 14.83	74.63 ± 14.07	*P* = 0.001	ns	*P* = 0.032
Height (cm)	171.17 ± 10.19	171.01 ± 9.25	173.05 ± 9.25	ns	ns	ns
BMI (kg/m2)	21.51 ± 3.72	23.29 ± 4.28	24.79 ± 3.51	*P* < 0.0001	ns	*P* = 0.004
L2-L4 BMD (g/cm2)	1.11 ± 0.18	1.16 ± 0.14	1.23 ± 0.08	*P* = 0.001	*P* = 0.019	ns
L2-L4 T-score	−0.90 ± 1.45	−0.42 ± 1.15	0.12 ± 0.69	*P* = 0.0006	*P* = 0.015	ns
L2-L4 Z-score	−0.12 ± 1.18	−0.12 ± 1.18	0.09 ± 0.64	*P* = 0.015	ns	ns
FN BMD (g/cm2)	0.94 ± 0.18	0.98 ± 1.18	1.08 ± 1.16	*P* = 0.0007	*P* = 0.010	ns
FN T-score	−0.64 ± 1.30	−0.31 ± 1.22	0.44 ± 1.02	*P* < 0.0001	*P* = 0.003	ns
FN Z-score	−0.25 ± 1.11	0.08 ± 1.06	0.38 ± 0.97	*P* = 0.006	ns	ns
sOPG (pmol/l)	8.65 ± 3.68	6.26 ± 2.56	9.59 ± 2.11	ns	*P* < 0.0001	*P* < 0.0001
sRANKL (pmol/l)	297.24 ± 197.76	228.21 ± 222.88	236.49 ± 110.69	ns	ns	*P* = 0.013
sOPG/sRANKL ratio	0.050 ± 0.053	0.046 ± 0.051	0.051 ± 0.029	ns	*P* = 0.042	ns
Vit. D_3_ (ng/ml)	22.29 ± 9.39	21.33 ± 12.20	21.56 ± 9.11	ns	ns	ns
Calcium (mmol/l)	2.32 ± 0.20	2.36 ± 0.14	2.38 ± 0.09	ns	ns	ns
IL-4 (pg/ml)	0.07 ± 0.12	0.27 ± 0.35	0.03 ± 0.03	ns	*P* < 0.0001	*P* < 0.0001
IL-10 (pg/ml)	1.22 ± 2.59	2.79 ± 3.99	0.81 ± 1.70	*P* = 0.014	*P* < 0.0001	*P* < 0.0001

All results are presented as means with standard deviations (SD)

*CD* Crohn’s disease, *UC* ulcerative colitis, *C* control group, *FN* femoral neck, *BMD* bone mineral density, *ns* non-significant

The analysis of the interleukin 4 and 10 levels showed increased concentrations in both CD and UC patients. However, in UC patients the IL-4 concentrations were increased over eightfold as compared to controls (0.272 vs 0.033 pg/ml, *P* value <0.0001), whereas in CD patients the increase was only twofold (0.070 vs 0.033 pg/ml) and this difference was not statistically significant. Similarly, average IL-10 concentrations in UC patients were over threefold higher than in healthy controls (2.792 vs 0.813 pg/ml, *P* value <0.0001), whereas in CD patients they were higher by half-fold (1.217 vs 0.813 pg/ml, *P* value = 0.0136).

The analysis of the rs2073617 polymorphism in the *TNFRSF11B* gene showed that the c.-223T allele was more frequent in IBD patients, with the odds ratio of 1.49. However, this result was not statistically significant (Table 2). The genotype distribution among all IBD patients together and among controls was concordant with the Hardy–Weinberg equilibrium. However, it was discordant when testing the groups of CD and UC patients separately.

**Table 2:** Alleles and genotypes distribution in rs2073617 *locus* in subjected patients with inflammatory bowel disease (IBD) and in control group

	*TNFRSF11B* c.-223C > T				
	Genotypic frequencies (%)			Allelic frequencies (%)	
	TT	CT	CC	T	C
IBD, all patients	69 (34.8 %)	94 (47.5 %)	35 (17.7 %)	232 (58.6 %)	164 (41.4 %)
Control group (*n* = 41)	9 (21.9 %)	22 (53.6 %)	10 (24.3 %)	40 (48.8 %)	42 (51.2 %)
IBD versus C OR (C.I.) *P* value	[TT] versus [CC]	[TT + CT] versus [CC]	[CT + CC] versus [TT]	[T] versus [C]	
	2.19 (0.815–5.884)	1.50 (0.674–3.346)	0.526 (0.237–1.165)	1.49 (0.922–2.393)	
	*P* = 0.114	*P* = 0.317	*P* = 0.109	*P* = 0.103	
UC patients (*n* = 98)	21 (21.4 %)	59 (60.2 %)	18 (18.4 %)	101 (51.5 %)	95 (48.5 %)
UC versus C OR (C.I.) *P* value	[TT] versus [CC]	[TT + CT] versus [CC]	[CT + CC] versus [TT]	[T] versus [C]	
	1.30 (0.432–3.890)	1.43 (0.596–3.447)	1.03 (0.462–2.494)	1.12 (0.667–1.870)	
	*P* = 0.64	*P* = 0.42	*P* = 0.95	*P* = 0.68	
CD patients (*n* = 100)	48 (48 %)	35 (35 %)	17 (17 %)	131 (65.5 %)	69 (34.5 %)
CD versus C OR (C.I.) *P* value	[TT] versus [CC]	[TT + CT] versus [CC]	[CT + CC] versus [TT]	[T] versus [C]	
	**3.14 (**1.090–9.027**)**	1.58 (0.651–3.810)	**0.31 (**0.132–0.704**)**	**1.99 (**1.183–3.360**)**	
	***P*** **=** **0.030**	*P* = 0.311	***P*** **=** **0.004**	***P*** **=** **0.009**	
CD versus UC OR (C.I.) *P* value	[TT] versus [CC]	[TT + CT] versus [CC]	[CT + CC] versus [TT]	[T] versus [C]	
	**2.42 (**1.047–5.595**)**	1.10 (0.529–2.281)	**0.30 (**0.159–0.550**)**	**1.79 (**1.192–2.676**).**	
	***P*** **=** **0.037**	*P* = 0.801	***P*** **<** **0.0001**	***P*** **=** **0.005**	

In bold: statistically significant results

*OR* odds ratio, *C.I*. 95 % confidence interval, *IBD* inflammatory bowel disease, *UC* ulcerative colitis, *CD* Crohn’s disease, *C* control group

There were no differences in the allele distributions between the UC patients and controls (OR = 1.12, C.I. = [0.667–1.870], *P* value = 0.68). However, among the CD patients, the c.-223T allele was two times more frequent than in controls and this result was statistically significant (OR = 1.99, C.I. = [1.183–3.360], *P* value = 0.009). The TT genotype was found in 48 % of CD patients, whereas among controls, it was found only in 21.9 % [OR = 3.14, C.I. = [1.090–9.027], *P* value = 0.03). Statistically significant differences in the allele and genotype distributions were also found between CD and UC patients (Table 2). A subsequent analysis of associations between the bone mass parameters (BMD, T-score and Z-score) and particular genotypes in *locus* rs2073617 gave only one statistically significant observation: the average lumbar spine Z-score which was twofold lower in TC heterozygotes as compared to TT homozygotes among CD patients (−1.27 vs −0.64, *P* value = 0.047).

The analysis of possible correlations of c.-223C > T polymorphism genotypes with fractures, serum RANKL levels and OPG/RANKL ratios did not show any statistically significant association. We also analysed possible relationship between the duration of the disease, the number of exacerbations, the number of hospitalisations and the location of the disease, and the polymorphic variants under study, but we did not find any statistically significant associations neither in CD nor in UC patients.

We did not confirm an unequivocal association of the c.-223T allele with lowered OPG levels. Only among CD patients, the TT genotype was correlated with a decreased level of osteoprotegerin in blood serum (OR = 3.28, C.I. = [1.421–7.581], *P* value = 0.004), whereas in UC patients this genotype had even a protective effect and was associated with increased OPG levels in this group (OR = 0.30, C.I. = [0.159–0.550], *P* value <0.0001).

## Discussion

Osteoporosis in inflammatory bowel disease (IBD) patients is a particularly important problem due to dangerous fracture complications that lead to immobilisation, muscular atrophy, bedsores, pulmonary embolisms and increased susceptibility to infections. These complications, particularly in elderly people, are a frequent cause of death. So far, the increased incidence of osteoporosis in IBD patients has been associated with undernutrition due to the disease itself and due to steroid treatments. However, recent data suggest that also genetic factors play a role [2]. In our study subjects, such parameters as vitamin D and calcium levels were not different between IBD patients and healthy controls. This means that the patients were not undernourished, and so it allows to confirm the hypothesis that osteoporosis in IBD patients is not related to low vitamin D or mineral elements supply and that seeking genetic factors is justified.

Both osteoporosis and IBD are multigenetic diseases, and a huge role in their pathogenesis is played by factors related to the inflammation process. However, no common susceptibility genes have been described so far. Functional polymorphisms in the *TNFRSF11B* gene coding for osteoprotegerin (OPG)—a protein with a function of a cytokine and a bone metabolism regulator—have appeared to us a promising research target. In this study, we analysed the frequency of the c.-223C > T (rs2073617) polymorphism in the *TNFRSF11B* gene in 198 IBD patients with a coexisting deviation from normal bone mineral density and in 41 healthy controls. We also tried to determine possible correlations of the different genotypes with the sOPG and sRANKL serum levels and bone mineral density.

Our study has also several limitations. The most important one is that we did not perform the association analysis between investigated genetic polymorphisms and the severity of the disease flare-up. Nevertheless, since all patients enrolled in the study had active disease, we decided not to divide the study group into clinical subcategories and not to reduce the power of the study. With the current study design, the study power was 80 % with a 10 % probability of type I error. The relatively low number of subjects is indeed a disadvantage of our study; however, we were limited by the prevalence of coexisting IBD and osteopenia or osteoporosis.

In Crohn’s disease (CD) patients, we observed the lowest values of bone mineral density (BMD), T-scores and Z-scores for both L2-L4 lumbar spine and femoral neck, which may indicate that bone tissue destruction processes in these patients are far more advanced and aggressive than in UC patients.

Among our study subjects, only in CD patients we observed lower sOPG levels in TT homozygotes as compared to carriers of CT and CC genotypes. The TT genotype in this group was three times more frequent than among controls and over two times more frequent than in UC patients. Taking into consideration other studies which have shown that the c.-223T allele is associated with lower serum OPG levels and predisposes to osteoporosis, our results may suggest that being a TT homozygote increases the risk of osteoporosis threefold, but only in CD patients.

Similar results on the association of the c.-223T allele (rs2073617) in the *TNFRSF11B* gene and lower BMD were reported by Vidal et al. [14]. In an in vitro experiment, they observed statistically significant differences in the *TNFRSF11B* gene expression levels depending on the c.-223C > T polymorphism. The c.-223T was associated with a lower expression of osteoprotegerin. As the latter has an anti-resorption function, this may result in increased resorption of bone tissue and thus the development of osteoporosis in the carriers of this allele [25]. The studies in Maltese and Dannish populations showed that the TT genotype was associated with lower BMD than other genotypes [25, 29]. Similarly, among Japanese post-menopausal women, TT homozygotes had significantly lower BMD than carriers of other genotypes and the C allele may have had a protective effect against the development of osteoporosis [30]. Results of our own unpublished pilot studies also suggested that the TT genotype is associated with the lowest average BMD. However, the authors of another study on a Japanese population have reported the contrary association: it was the CC genotype that correlated with lower BMD of the wrist as compared to the TT and TC genotypes [31]. There are also studies which did not show an association of any of the *TNFRSF11B* c.-223C > T polymorphic alleles with osteoporosis—these were conducted on an Irish, Slovenian, Dannish, Swedish and Australian population [27, 29, 32–34]. Also a meta-analysis of eight studies that were conducted on different European and Asian populations did not show any association between the polymorphism under study and BMD of femoral neck and lumbar spine [35]. The divergence of results obtained in studies of different populations suggests that the c.-223C > T polymorphism may be non-functional. However, it is possible that this polymorphism may be in linkage disequilibrium with an unknown functional variant of the *TNFRSF11B* gene and modulate its expression by affecting the mRNA stability. It is also possible that the coexistence of these two or the other variants has an additive effect and alters the minimal free energy of the transcript and the mRNA thermodynamic stability, which in turn may change the mRNA degradation levels.

In our UC patients and in controls, we did not observe a correlation between the TT genotype and lower osteoprotegerin levels, either. On the contrary, there was a trend for TT homozygotes having higher sOPG concentrations than carriers of the CC genotypes, but the difference was not statistically significant. UC patients, regardless of the genotype, had the lowest sOPG levels (*P* value <0.0001). It is possible that other alterations in the *TNFRSF11B* gene lead to its lower activity or the production of a non-functional protein. Moreover, the loci of genetic susceptibility to CD and UC overlap only to some extent and there are more and more loci known to be associated with the susceptibility to each of these diseases separately [36]. Thus, the susceptibility to osteoporosis in CD and UC patients may be determined by different genetic factors.

We think that research should focus also on various cytokines produced by lymphocytes as they play crucial roles in bone metabolism, either directly or through OPG. In addition to many common signalling molecules, T lymphocytes and bone cells have also a common origin: bone marrow. Studies on animals and on women with post-menopausal osteoporosis have shown that loss of the bone mass is correlated with an increased T lymphocyte activity as compared to healthy post-menopausal women [37]. The RANK/RANKL/OPG pathway is also involved in interactions between T lymphocytes and dendritic cells. OPG reduces the survival of dendritic cells, regulates T lymphocytes activation, as well as B lymphocytes maturation and functions [38]. Immature dendritic cells excrete IL-10 that, in turn, regulates processes related to immune tolerance. Studies on mice have shown that RANK-induced stimulation may enhance both the immunotolerant capacities of the dendritic cells in physiological conditions and their immunogenicity in inflammatory tissue, probably by affecting the survival of dendritic cells [39]. Among our study subjects, IL-10 levels in UC patients were three times higher and in CD patients half a time higher than in controls. Given the low osteoprotegerin levels in UC patients, high IL-10 concentrations in these individuals may result from a lowered OPG expression.

Interleukin-4 (IL-4) levels in our UC patients were increased multifold (over eight times). IL-4 is a pleiotropic cytokine produced by T lymphocytes, mastocytes, basophilic granulocytes and natural killer (NK) cells, which induces OPG production by osteoblasts and thus inhibits osteoclast activity [40]. Thus, in theory, these patients should have increased OPG levels in sera. Yet, we found that the average OPG concentration in UC patients was significantly lower than in controls (*P* value <0.0001). In CD patients, OPG levels were only slightly lower and IL-4 concentrations were on average twice higher than in controls, but this was not statistically significant.

In summary, the results of our genetic analyses did not allow to define an unequivocal association of the *TNFRSF11B* c.-223C > T polymorphism and the blood serum osteoprotegerin levels. The c.-223T allele was significantly more frequent in CD patients than in UC patients (*P* value = 0.037) and in controls (*P* value = 0.030). It should be emphasised that the CD patients had the highest sRANKL levels and the lowest bone mineral density of both femoral neck and lumbar spine (L2-L4). This is in accordance with the results of Franke et al. [9] who pointed out to the *RANKL* gene as a new quantitative trait locus (QTL) for susceptibility to the Crohn’s disease and associated osteoporosis [41].

We conclude that low OPG levels may be associated with osteoporosis in ulcerative colitis patients, but not necessarily in CD patients. The differences in OPG, RANKL, IL-4 and IL-10 levels we observed between CD and UC patients confirm a different pathophysiology of these two diseases and a different molecular background of coexisting osteoporosis. Further studies should focus not only on the RANK/RANKL/OPG pathway genes but also on interleukins genes and this in larger study groups.
